# Cell Cycle-Dependence of Autophagic Activity and Inhibition of Autophagosome Formation at M Phase in Tobacco BY-2 Cells

**DOI:** 10.3390/ijms21239166

**Published:** 2020-12-01

**Authors:** Shigeru Hanamata, Takamitsu Kurusu, Kazuyuki Kuchitsu

**Affiliations:** 1 Department of Applied Biological Science, Tokyo University of Science, 2641 Yamazaki, Noda, Chiba 278-8510, Japan; hanamata@agr.niigata-u.ac.jp; 2 Graduate School of Science and Technology, Niigata University, 2-8050 Ikarashi, Niigata, Nishi-ku 950-2181, Japan; 3 Department of Mechanical and Electrical Engineering, Suwa University of Science, 5000-1 Toyohira, Chino, Nagano 391-0292, Japan

**Keywords:** ATG8, autophagy, cell cycle, tobacco bright yellow-2 (BY-2) cells

## Abstract

Autophagy is ubiquitous in eukaryotic cells and plays an essential role in stress adaptation and development by recycling nutrients and maintaining cellular homeostasis. However, the dynamics and regulatory mechanisms of autophagosome formation during the cell cycle in plant cells remain poorly elucidated. We here analyzed the number of autophagosomes during cell cycle progression in synchronized tobacco BY-2 cells expressing YFP-NtATG8a as a marker for the autophagosomes. Autophagosomes were abundant in the G2 and G1 phases of interphase, though they were much less abundant in the M and S phases. Autophagosomes drastically decreased during the G2/M transition, and the CDK inhibitor roscovitine inhibited the G2/M transition and the decrease in autophagosomes. Autophagosomes were rapidly increased by a proteasome inhibitor, MG-132. MG-132-induced autophagosome formation was also markedly lower in the M phases than during interphase. These results indicate that the activity of autophagosome formation is differently regulated at each cell cycle stage, which is strongly suppressed during mitosis.

## 1. Introduction

Autophagy, an evolutionarily conserved system for the bulk degradation and recycling of nutrients, is induced in many eukaryotes, including plants, by the limitation of various nutrients or under biotic and abiotic stresses and at specific developmental stages. Such inducible bulk-recycling autophagy plays important roles in cell survival and the maintenance of growth, as well as development and stress adaptation [1,2,3,4]. In addition to bulk autophagy, selective autophagy behaves as a very specific process for the removal of target organelles and molecules [5,6,7]. Autophagy is also constitutively maintained at low levels in most cells and plays important roles in cellular homeostasis under normal growth conditions [8,9,10,11]. In plants, such constitutive autophagy has also been observed in root tissues in several plant species without nutrient starvation, and plays a role in root cell growth and lipid metabolism under normal growth conditions [12,13,14].

In animal and yeast cells, autophagy is closely linked to cell cycle-related development, such as cell differentiation, programmed cell death, as well as cell division [15,16,17]. In animal cells, autophagy is induced preferentially in the G1 and S phases of the cell cycle by various autophagy activators and starvation, and is inhibited during mitosis [18,19,20]. Starvation-induced autophagy is known to be crucially involved in the cell cycle progression from the G2/M to the G1 phase in yeast [21].

The possible involvement of autophagy in cell differentiation and programmed cell death (PCD) has also been discussed in plants [22]. Autophagy is suggested to contribute to tracheary element differentiation in *Arabidopsis* [23,24] and PCD in the embryo suspensor of Norway spruce [25]. Autophagy is reported to regulate hypersensitive response (HR)-PCD positively in young plants and negatively in old plants [26]. Moreover, autophagy is involved in PCD and lipid metabolism during pollen maturation in rice anther tapetum cells [27,28]. However, despite the close connection between cell differentiation or PCD and the cell cycle, the dynamics and regulatory mechanisms of autophagy during the cell cycle in plant cells remain mostly unknown.

Tobacco BY-2 cells are especially advantageous in highly synchronizing the cell cycle and thereby studying intracellular localization and the dynamics of proteins and organelles [29,30]. The connection between the cell cycle and stress-induced PCD has been studied [31,32], and in vivo quantitative monitoring systems for autophagic flux have recently been established [33]. Therefore, BY-2 cells are useful for understanding autophagy dynamics in the cell cycle of plant cells.

In this study, we analyzed the formation of autophagosomes during cell cycle progression in synchronized tobacco BY-2 cells expressing the YFP-NtATG8a fusion protein as a marker for the autophagosomes [33]. Pharmacological analysis and in vivo imaging revealed that autophagy was differently regulated at each phase during cell cycle progression, and the number of autophagosomes increased during interphase and was strongly suppressed during mitosis in tobacco BY-2 cells.

## 2. Results and Discussion

### 2.1. Fluctuation of Autophagosome Formation during Cell Cycle Progression

**Figure 1:**
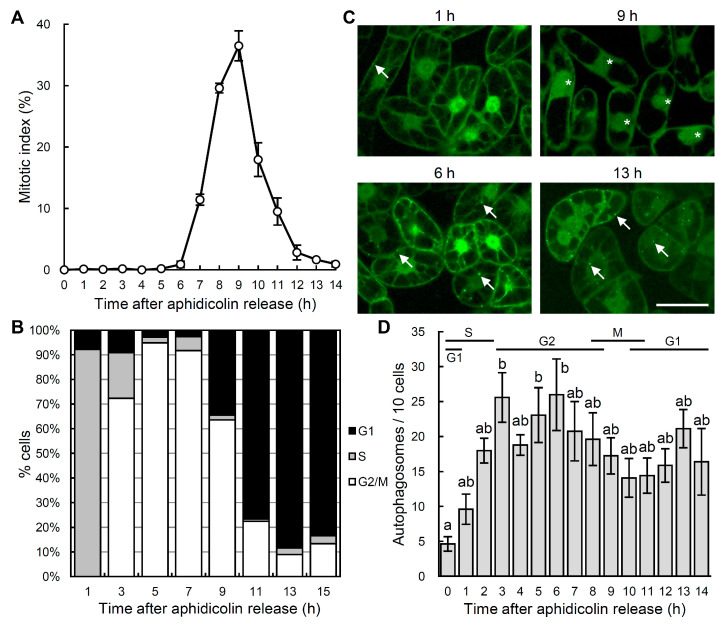
Synchronization of the cell cycle and visualization of the dynamics of autophagosome formation in tobacco BY-2 cells using aphidicolin. The cell cycle of seven-day-old BY-YA8 cells was synchronized at the S phase using aphidicolin for 24 h. Cells were then released from the aphidicolin block and incubated for 14 h. (**A**) Monitoring of the mitotic index during cell cycle progression in BY-YA8 cells. Data are the means ± SE of three independent experiments. (**B**) Progression of the cell cycle was monitored using flow cytometry. Data are representative of three experiments. (**C**) Images were obtained by confocal laser scanning microscopy. Arrows and asterisks indicate punctate signals of YFP-NtATG8a that correspond to the autophagosomes [33] and mitotic cells, respectively. Scale bars: 50 μm. Data are representative of three experiments. (**D**) The levels of autophagy at each phase of the cell cycle in BY-2 cells. To quantify the levels of autophagosome formation, the number of YFP punctate signals per 10 cells was quantified at the indicated time points. Data are the means ± SE of three independent experiments. a, b: values with different letters are significantly different (*p* < 0.05).

We used the transgenic tobacco BY-2 cell line (BY-YA8), constitutively over-expressing the YFP-NtATG8a fusion protein, under the control of cauliflower mosaic virus (CaMV) 35S promoter, to visualize the autophagosomes [33]. To analyze the dynamics of autophagosome formation during cell cycle progression, a 7-day-old BY-YA8 suspension culture was synchronized using aphidicolin treatment. After releasing the cells from the aphidicolin block, cell cycle progression was monitored by both determining the mitotic index and by flow cytometric analysis (Figure 1A,B). The mitotic index, which indicates the proportion of cells in the M phase, increased from 7 h after the aphidicolin release, peaked at approximately 40% 9 h after release, and then decreased, which is consistent with the previous literature studying cell-cycle-specific events in tobacco BY-2 cells [31,32]. Flow cytometric analysis revealed that more than 90% of the cells were in the S phase 1 h after the aphidicolin release, and 90% of the cells were in the G2 phase 5 h after the release (Figure 1B). Therefore, cell cycle stages were highly synchronized for at least 5 h after the aphidicolin release.

The number of autophagosomes was quantified using a confocal laser scanning microscope (Figure 1C,D). Only a few autophagosomes were detected 0 h after the aphidicolin release, when more than 90% of the cells were at the S phase. The number of autophagosomes subsequently significantly increased from 3 h to 6 h after the release, when 70–90% of the cells corresponded to the G2 phase. It reached a plateau and did not significantly change until 14 h after the release. These results suggest that during cell cycle progression from the S phase to the G2 phase, either the enhancement of autophagosome formation or the suppression of autophagosomal fusion with the vacuole is induced (Figure 1).

**Figure 2:**
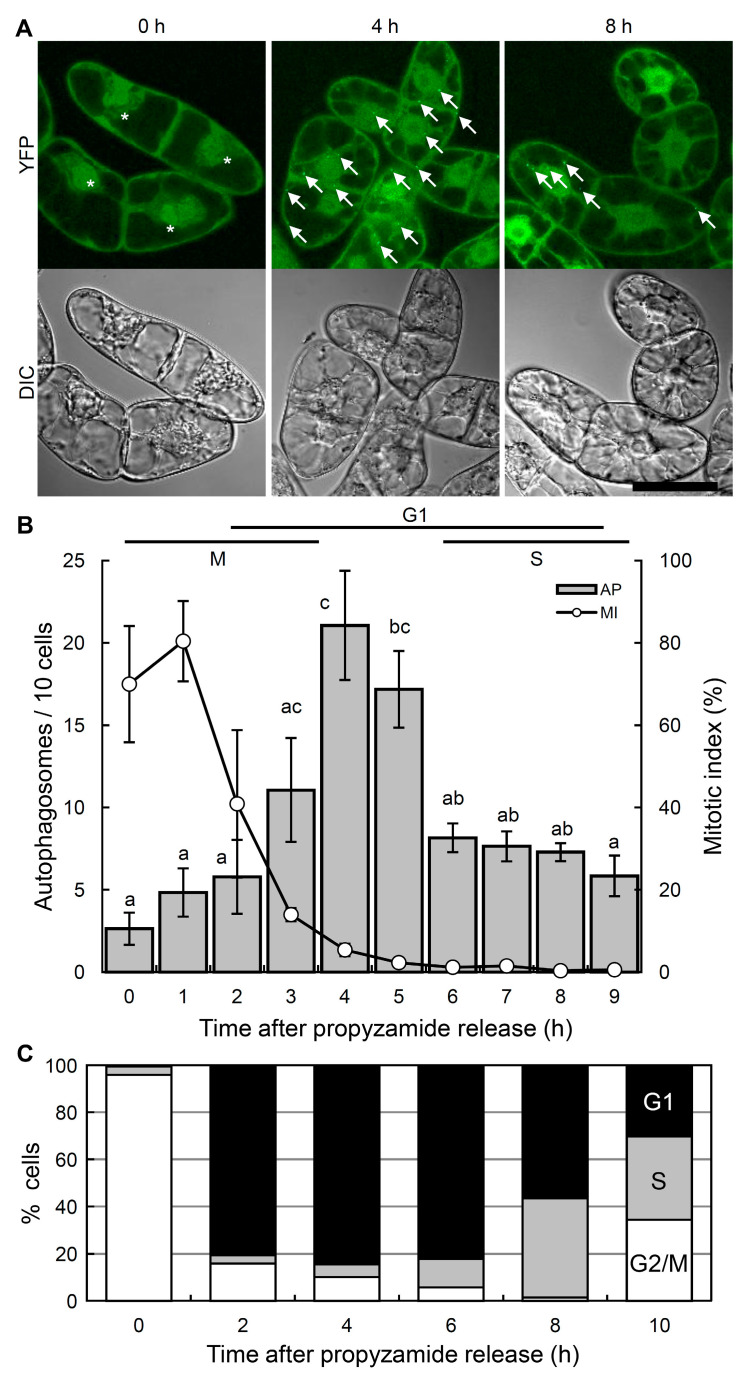
Changes in the number of autophagosomes during cell cycle progression in synchronized BY-2 cells. The cell cycle of seven-day-old BY-YA8 cells were synchronized at the M phase by the aphidicolin/propyzamide double synchronization method. (**A**) Fluorescence (upper) and differential interference contrast (DIC) images (lower) were obtained by confocal laser scanning microscopy. Arrows and asterisks indicate punctate signals of YFP-NtATG8a that correspond to the autophagosomes [33] and mitotic cells, respectively. Scale bars: 50 μm. Data are representative of four experiments. (**B**) Time-course changes in the punctate signals of YFP-NtATG8a (bars) and mitotic index (open circles), respectively. To quantify the levels of autophagosome formation, the number of YFP punctate signals per 10 cells was quantified at the indicated time points. Data are the means ± SE of four independent experiments. a, b, c: values with different letters are significantly different (*p* < 0.05). AP: Autophagosome (**C**) Progression of the cell cycle was monitored by flow cytometry. Data are representative of four experiments.

To analyze the dynamics of autophagy after mitosis, we adopted the double synchronization method using aphidicolin and propyzamide [34] to further synchronize the cell cycle. The number of autophagosomes was quantified after the propyzamide release (Figure 2A,B). The sequential cell cycle block by aphidicolin and propyzamide resulted in a higher level of synchronization at the prometaphase, with the mitotic index reaching nearly 70% of the cells (Figure 2B, 0 h). The mitotic index peaked at approximately 80% 1 h after the propyzamide release, then rapidly dropped and remained at negligible levels until 9 h after the release (Figure 2B). Flow cytometric analysis revealed that 90% of cells were in the G2/M phase at the time of propyzamide release (Figure 2C, 0 h) and 85% in the G1 phase 4 h after the release (Figure 2C, 4 h).

A few autophagosomes were detected when cells were released from the propyzamide block, when 70% of the cells corresponded to the M phase (Figure 2B, 0 h). The number of autophagosomes subsequently increased and peaked approximately 4 h after the release from the propyzamide block, when 85% of the cells corresponded to the G1 phase, then subsequently decreased during the G1/S transition (Figure 2). Taken together, autophagosome formation is suppressed at the M phase, drastically activated at the G1 phase, and inhibited at the late G1 phase and the S phase.

**Figure 3:**
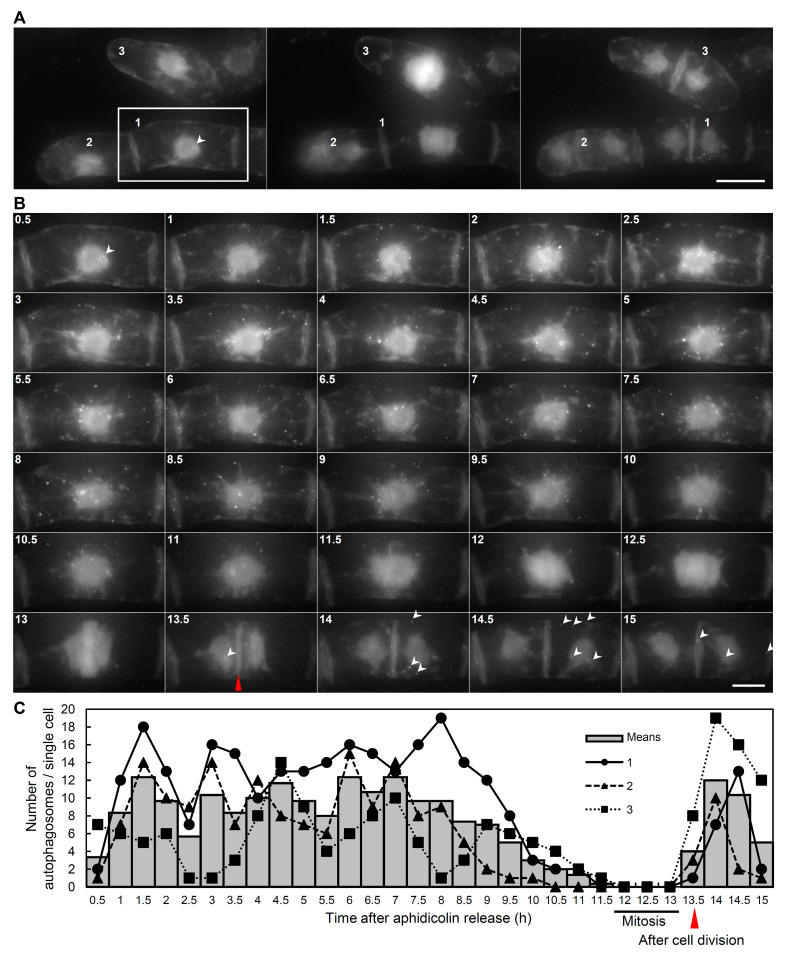
Time-lapse imaging of autophagosome formation during cell cycle progression in tobacco BY-2 cells. The cell cycle of seven-day-old BY-YA8 cells was synchronized at the S phase using aphidicolin for 24 h. Cells were then released from the aphidicolin block and incubated for 15 h on the glass bottom dish. (**A**,**B**) Images show maximum intensity Z-projections containing the whole cells obtained by fluorescence microscopy, and contain all autophagosomes of whole cells of tobacco BY-2. Arrowheads indicate the punctate signals of YFP-NtATG8a. Left, central, and right panels show cells before cell division, under mitosis, and after cell division, respectively. Scale bars: 30 μm. Data are representative of three experiments. (**B**) Time-lapse Z-stack imaging of single cell in (**A**). Images were taken every 30 min. Arrowheads and red triangle indicate punctate signals and results obtained after cell division, respectively. Scale bars: 15 μm. (**C**) The number of autophagosomes at each point of the cell cycle in tobacco BY-2 cells. To quantify the levels of autophagosome formation, the number of YFP punctate signals of a single cell was quantified at the indicated time points. Bars are the means of three independent cells in (**A**). The plots show the number of signals in each cell.

We further performed time-lapse imaging analysis to determine the spatiotemporal dynamics of the autophagosomes during the cell cycle progression in single cells (Figure 3 and Supplementary Movie 1). As shown in Figure 3, the punctate signals corresponding to the autophagosomes were abundant in the interphase (Figure 3B,C; 1.5–9.5 h). On the other hand, the number of punctate signals decreased before the cell division (Figure 3B,C; 10–11.5 h), reached the minimum level during mitosis (Figure 3B,C; 12–13 h), and increased again after the cell division (Figure 3B,C; 13.5–15 h). These results are consistent with the results shown in Figure 1 and Figure 2, indicating the decrement of autophagosomes during mitosis.

In order to confirm if chemicals used for the cell cycle synchronization affected autophagic activity, we tested the effects of aphidicolin and propyzamide on autophagosome formation (Supplementary Figure S1). Treatment of aphidicolin in 4-day-old BY-YA8 cells induced a slight increase in the number of autophagosomes. Propyzamide did not affect the number of autophagosomes. As a control, 3-methyladenine (3-MA), a phosphoinositide 3-kinase (PI3K) inhibitor that is known to inhibit autophagy, drastically reduced the number of autophagosomes. A salicylic acid analog benzo(1,2,3)thiadiazole-7-carboxylic acid (BTH) induced a strong increase in autophagosomes, which was suppressed by 3-MA, as described previously [33]. Neither aphidicolin nor propyzamide affected the BTH-induced increase in autophagosomes (Supplementary Figure S1), suggesting that these chemicals used for cell cycle synchronization do not affect the autophagic activity.

Overall, our results indicate that the number of autophagosomes fluctuates during cell cycle progression. In *Arabidopsis* root cells, the activity of constitutive autophagy is reported to be low in the meristematic zone and high in the cells in the elongated and differentiated zones [12]. Normal cell cycle progression occurs in the meristematic zone as in tobacco BY-2 cells, whereas an endocycle, cell cycle progression without mitosis, occurs in the elongated and differentiated zones in which most cells are in the G or S phases [35]. Active autophagy in the root cells in the elongated and differentiated zones may reflect the more active autophagosome formation in the G1 and G2 phases, as shown in the present study (Figure 1 and Figure 2). In an autophagy-defective mutant of *Arabidopsis*, root cells are shorter in length, with smaller vacuoles than the wild-type [36]. Therefore active autophagosome formation during interphase in tobacco BY-2 cells, as well as in the elongation and differentiated zones of root cells, may play an important role in the expansion of the vacuoles and cell elongation.

### 2.2. Decrease in Autophagosomes during the G2/M Transition

**Figure 4:**
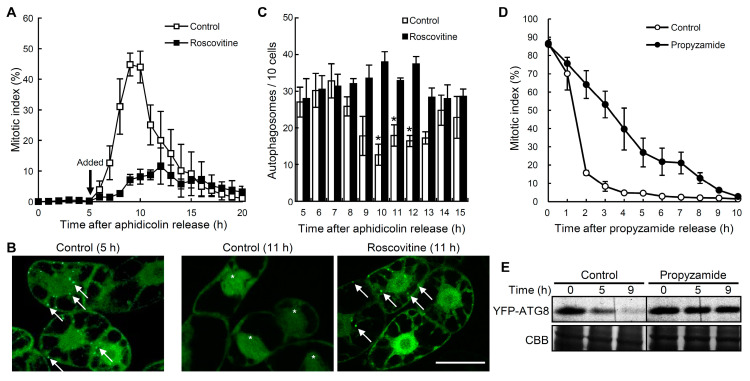
Reductions in the number of autophagosomes were dependent on cell cycle progression from the G2 phase to the M phase in tobacco BY-2 cells. BY-YA8 cells were synchronized at the S phase using aphidicolin treatment. Roscovitine (50 μM) was applied 5 h after release from the aphidicolin block. (**A**) Time-course graph of the mitotic index after aphidicolin was removed. Open and closed boxes indicate the absence and presence of roscovitine, respectively. Data are the means ± SE of three independent experiments. (**B**) Images were obtained by confocal laser scanning microscopy. The levels of autophagy at 5 h (G2 phase) and 11 h (M phase, control), respectively. Arrows indicate the punctate signals of YFP-NtATG8a. Asterisks indicate mitotic cells. Bars: 50 μm. (**C**) Quantitative levels of the number of punctate signals in (**B**). The number of YFP punctate signals per 10 cells was counted. Open and closed bars indicate the absence and presence of roscovitine, respectively. Data are the means ± SE of three independent experiments. * *p* < 0.05, significantly different from the control. (**D**) Changes in the mitotic index of non-released cells (black circles) and released cells (white circles). Data are the means ± SE of three experiment. (**E**) SDS-PAGE and fluorescence image analysis of released cells (Control) and non-released cells (Propyzamide). To detect the YFP fluorescence in the SDS-PAGE gels, unheated protein samples (20 μg per lane) were subjected to SDS-PAGE and the fluorescence of YFP-NtATG8a was detected using a 473-nm excitation laser through a 520-nm longpass emission filter, using the fluoroimage analyzer FLA3000G. Coomassie brilliant blue (CBB) staining was used as a loading control. Representative results of three independent experiments are shown.

As shown in Figure 3, the number of autophagosomes transiently decreased with the increase in the number of mitotic cells. Thus, we hypothesized that autophagic activity may change during cell cycle progression from the G2 phase to the M phase. To confirm this hypothesis, we analyzed the effect of a cyclin-dependent kinase (CDK) inhibitor roscovitine, which blocks the G2/M transition in tobacco BY-2 cells [37]. Five h after the release, which corresponded to the early G2 phase, aphidicolin-synchronized cells were incubated with or without roscovitine and cell cycle progression was monitored by determining the mitotic index (Figure 4A). Roscovitine inhibited an increase in the number of mitotic cells (Figure 4A) and blocked a decrement of autophagosomes (Figure 4B,C). During the cell cycle progression from the M phase to the G1 phase, we also observed a decrease in the fluorescent signals of the YFP-NtATG8a protein (Figure 4D,E), which is presumably attributed to the induction of autophagy [38]. These results indicate that a decrease in the number of autophagosomes is induced during the G2/M transition of the cell cycle, and the autophagic activity is regulated in a cell-cycle-dependent manner in tobacco BY-2 cells.

In animal cells, the numbers of autophagy-related structures were also shown to be reduced during mitosis [18]. The interaction between vacuolar protein sorting 34 (Vps34) and Beclin1, a component of the phosphoinositide 3-kinase (PI3K) complex necessary for the formation of autophagosomes, was recently shown to be inhibited by CDK during the M phase [20]. Thr159 of Vps34, an important phosphorylation site in the interaction between Vps34 and Beclin1, was found to be conserved in *Arabidopsis*, rice, and tobacco (Figure S2), suggesting that the inhibition of autophagy during mitosis shown for the first time in the present study in plants may also be due to a similar mechanism, and the regulatory mechanism of autophagy repression during mitosis may be highly conserved in animals and plants. The relationship between autophagy dynamics and the regulation of Vps34 phosphorylation status by CDK during cell cycle progression in plant cells should be an important future research topic.

### 2.3. MG-132-Induced Autophagosome Formation Is Differentially Regulated in Cell Cycle Stages and Suppressed at M Phase

**Figure 5:**
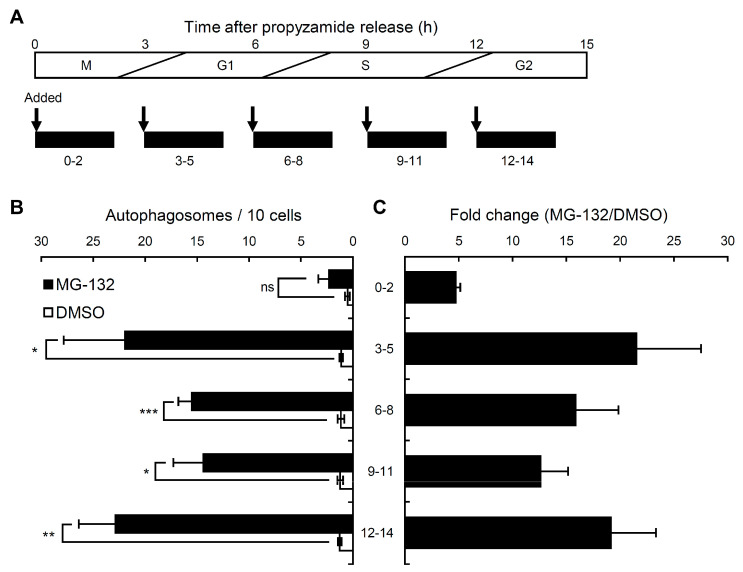
MG-132-inducible increments in the number of autophagosomes at each phase of the cell cycle. BY-YA8 cells were synchronized at the M phase using aphidicolin/propyzamide treatment. MG-132 (100 μM) was applied 0–2 h (M phase), 3–5 h (G1 phase), 6–8 h (G1–S phase), 9–11 h (S–G2 phase), and 12–14 h (G2 phase) after release from the propyzamide block. (**A**) Time-course graph of the treatment with MG-132 after propyzamide was removed. (**B**) The levels of autophagy at 2 h, 5 h, 8 h, 11 h, and 14 h, respectively. (**C**) The relative levels of autophagy were standardized with controls at each point as 1. DMSO was used as the control. Bars are the means ± SE of three independent experiments. * *p* < 0.05, ** *p* < 0.005, *** *p* < 0.001; significantly different from the controls.

Since the number of autophagosomes was specifically reduced in the M and S phase, we tested whether the level of inducible autophagy is also affected by the cell cycle phases. Cells were treated for 2 h with a proteasome inhibitor, MG-132, which has been shown to activate autophagy in both plants and animals [39,40]. Similarly, the number of autophagosomes increased rapidly with MG-132 treatment (100 μM) in non-synchronized tobacco BY-2 cells (Figure S3). As shown in Figure 5, the MG-132 treatment induced an increase in autophagosomes in synchronized tobacco BY-2 cells (Figure 5B). The increase at 0–2 h after the propyzamide release was significantly weaker than that at 3–5 h (80% of the cells were in the G1 phase, Figure 2C), 6–8 h (most cells were in the G1 and S phases, Figure 2C), 9–11 h (most cells were in the S and G2 phases, Figure 2C), and 12–14 h (50% of the cells were in the G2 phase), indicating that MG-132-induced autophagosome formation is differentially regulated in cell cycle stages and lowest at the M phase. These results suggest that the cellular potential to induce autophagy is affected by the cell cycle, and is specifically suppressed at the M phase. Repression of autophagy during mitosis may be an important means of protecting mitotic machinery from autophagic degradation.

The proteasome inhibitor MG-132 rapidly induced autophagy in plant cells (Figure 5), which is consistent with observations in animal cells [39]. Though its mechanism has not been elucidated, this may be due to the complementary regulation of the degradation of ubiquitinated proteins by the proteasome and autophagy. Various ubiquitinated proteins, including cell cycle regulators, have recently been shown to be degraded not only by the ubiquitin-proteasome system, but also by selective autophagy in animals and plants [5,41,42,43], suggesting that degradation of the cell cycle regulators by autophagy may be involved in the cell cycle regulation. Detailed analyses of the cell cycle progression and the degradation of ubiquitinated proteins in autophagy-defective plant cells may provide a novel insight on the roles of autophagy in cell cycle regulation.

## 3. Materials and Methods

### 3.1. Cell Culture, Growth Conditions, and Synchronization

A tobacco BY-2 (*Nicotiana tabacum* L. cv. Bright Yellow 2) cell suspension was maintained through the weekly dilution (1/100) of cells in modified Linsmaier and Skoog (LS) medium, as described [44]. The cell suspension was agitated on a rotary shaker at 95 rpm at 25 °C in the dark. A transgenic BY-2 cell line stably expressing *YFP-NtATG8a* (BY-YA8 cells) was established as described [33].

Synchronization of the cell cycle at the S phase was performed as described [34]. A stationary culture of BY-YA8 cells was diluted 1/10 in fresh modified LS medium supplemented with 5 µg/mL aphidicolin (FUJIFILM Wako Pure Chemical, Osaka, Japan). After culturing for 24 h, aphidicolin was removed by extensive washing and the cells were resuspended in fresh medium. To specify the M phase by sequential synchronization, the cells were treated with 3 μM propyzamide (FUJIFILM Wako Pure Chemical, Osaka, Japan) 5 h after the removal of propyzamide [34].

### 3.2. Monitoring the Cell Cycle

To measure the mitotic index, the nuclei of the cells were stained with SYTOX (Thermo Fisher Scientific, Waltham, MA, USA). An aliquot of the cell suspension (10 mg fresh weight in 0.1 mL) was incubated with 1 mg/mL SYTOX for 10 min. The mitotic index was quantified by counting dividing cells (250–300 cells per each sample) using a fluorescence microscope [45].

Flow cytometric analysis was performed as described [45]. An aliquot of the cell suspension was collected using a filter flask. A sample of a frozen cell pellet was treated using the CyStain UV precise P kit (Sysmex, Hyogo, Japan) to determine the DNA content. To release cell nuclei, the cells were chopped with a sharp razor blade in 400 μL extraction buffer and filtered through a 100-μm nylon filter. The separated nuclei were stained with 800 μL staining buffer. The fluorescence intensity was measured using a Ploidy Analyzer (CyFlow;Sysmex, Hyogo, Japan).

### 3.3. Confocal Imaging and Quantification of Autophagosomes

Images were taken with a 40× objective lens on a microscope (AXIOobserver.Z1; Carl Zeiss, Jena, Germany) equipped with a confocal laser scanning head and control systems (LSM5 EXCITER; Carl Zeiss, Jena, Germany). In all experiments, laser intensity was adjusted to the lowest level that retained a significant signal-to-noise ratio. To count autophagosomes, transgenic tobacco BY-2 cells were mounted onto glass slides, the punctate fluorescence signals of YFP in one slice in the confocal image through the center of the cells were counted for at least 200 cells, and the average was determined for 10 cells per each treatment, as described [33].

Time-lapse imaging of autophagosome formation during the cell cycle in tobacco BY-2 cells was taken using an all-in-one fluorescence microscope (BZ-9000; Keyence, Osaka, Japan). In all experiments, exposure time was adjusted to the shortest level that retained a significant signal-to noise ratio. To count autophagosomes, transgenic tobacco BY-2 cells were fixed onto a glass bottom dish using poly-L-lysine. The punctate fluorescence signals of YFP in maximum intensity Z-projections in the Z-Stack images in the whole cell were counted in the single cell.

### 3.4. SDS-PAGE and Fluorescence Image Analysis

SDS-PAGE and fluorescence image analysis was performed as described [38]. An aliquot of the cell suspension was collected using a filter flask. Crude protein samples were extracted with protein extraction buffer (50 mM MOPS-KOH, 2.5 mM EDTA2Na, 100 mM NaCl, 0.05 % (*w*/*v*) TritonX-100, pH 7.5). To detect the YFP fluorescence in the SDS-PAGE gels, unheated protein samples (20 μg per lane) were subjected to SDS-PAGE and the fluorescence of YFP-NtATG8a was detected using a 473 nm excitation laser through a 520 nm longpass emission filter, using the fluoroimage analyzer FLA3000G (GE Healthcare Bio-sciences, Piscataway, CA, USA).

### 3.5. Statistical Analysis

Statistical significance was determined using an unpaired Student’s *t*-test and ANOVA (analysis of variance) followed by a pairwise comparison test using Welch’s *F*-test in one-way ANOVA, not assuming equal variances, using R software (version 4.0.3) and an R commander (version 2.7-1).

## 4. Conclusions

**Figure 6:**
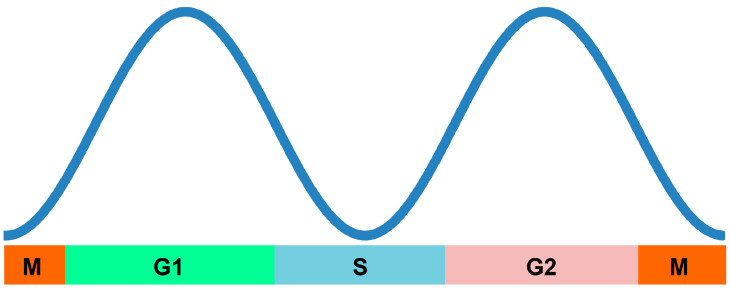
A schematic representation of the regulation of autophagosome formation during the cell cycle in BY-2 cells. The number of autophagosomes dynamically fluctuated during cell cycle progression; low in the M and S phases and high in the G1 and G2 phases. It decreases during G2/M and G1/S transitions. MG-132-induced autophagy was also suppressed at the M phase.

The present study demonstrates that the number of autophagosomes fluctuates during the cell cycle and is low at M and S phases, yet high in G1 and G2 phases of interphase (Figure 6). The potential to induce autophagy, at least that triggered by MG-132 treatment, was also suppressed at the M phase. We propose that fluctuations in autophagy at each phase of the cell cycle may be involved not only in survival under starvation conditions, but also in normal growth in plant cells. These findings shed light on novel aspects of the physiological functions of basal autophagy in cell cycle progression, as well as cell proliferation in plant cells. The cell cycle’s regulatory machinery and autophagy may crosstalk and regulate each other. The molecular mechanisms underlying such crosstalk should be an important future research topic.

## Supplementary Materials

Supplementary materials can be found at https://www.mdpi.com/1422-0067/21/23/9166/s1. Supplementary Movie 1: Time-lapse imaging of autophagosome formation during cell cycle progression in tobacco BY-2 cells. Supplementary Figure S1: The effect of aphidicolin/propyzamide on autophagosome formation in tobacco BY-2 cells. Supplementary Figure S2: Amino acid sequence alignment of plant Vps34 proteins with Vps34 of *Homo sapiens*. Supplementary Figure S3: The effect of MG-132 on autophagosome formation in tobacco BY-2 cells.

## Abbreviations

ATG	Autophagy
BY-2	Bright yellow-2
BY-YA8	Transgenic tobacco BY-2 cell line (BY-YA8) stably expressing a YFP-NtATG8a fusion protein
CLSM	Confocal laser scanning microscopy
DIC	Differential interference contrast
GFP	Green fluorescent protein
LS	Linsmaier and Skoog
MG-132	Carbobenzoxyl-leucinyl-leucinyl-leucinal
PI3K	Phosphoinositide 3-kinase
YFP	Yellow colored variant of GFP
