# Supplementary material for: Cell Cycle-Dependence of Autophagic Activity and Inhibition of Autophagosome Formation at M Phase in Tobacco BY-2 Cells
Source: Int J Mol Sci. 2020 Dec 1;21(23):9166. doi: 10.3390/ijms21239166 (PMC7730373; doi:10.3390/ijms21239166)
Supplement: Supplementary file 1 [file ijms-21-09166-s001.zip › ijms-1018172 supplementary done/ijms--supplementary.pdf]

Supplementary Materials for:

Cell Cycle-Dependence of Autophagic Activity and Inhibition of Autophagosome Formation at M Phase in Tobacco BY-2 cells

Shigeru Hanamata [1,2], Takamitsu Kurusu [1,3,*] and Kazuyuki Kuchitsu [1,*]

[1] Department of Applied Biological Science, Tokyo University of Science, 2641 Yamazaki, Noda, Chiba 278-8510, Japan; kuchitsu@rs.tus.ac.jp
[2] Graduate School of Science and Technology, Niigata University, 2-8050 Ikarashi, Niigata, 950-2181, Japan; hanamata@agr.niigata-u.ac.jp
[3] Department of Mechanical and Electrical Engineering, Suwa University of Science, 5000-1 Toyohira, Chino, Nagano, 391-0292, Japan; kurusu@rs.sus.ac.jp
* Correspondence: kurusu@rs.sus.ac.jp (T.K.); kuchitsu@rs.tus.ac.jp (K.K.); Tel.: +81-266-73-9826 (T.K.); +81-4-7122-9404 (K.K.)

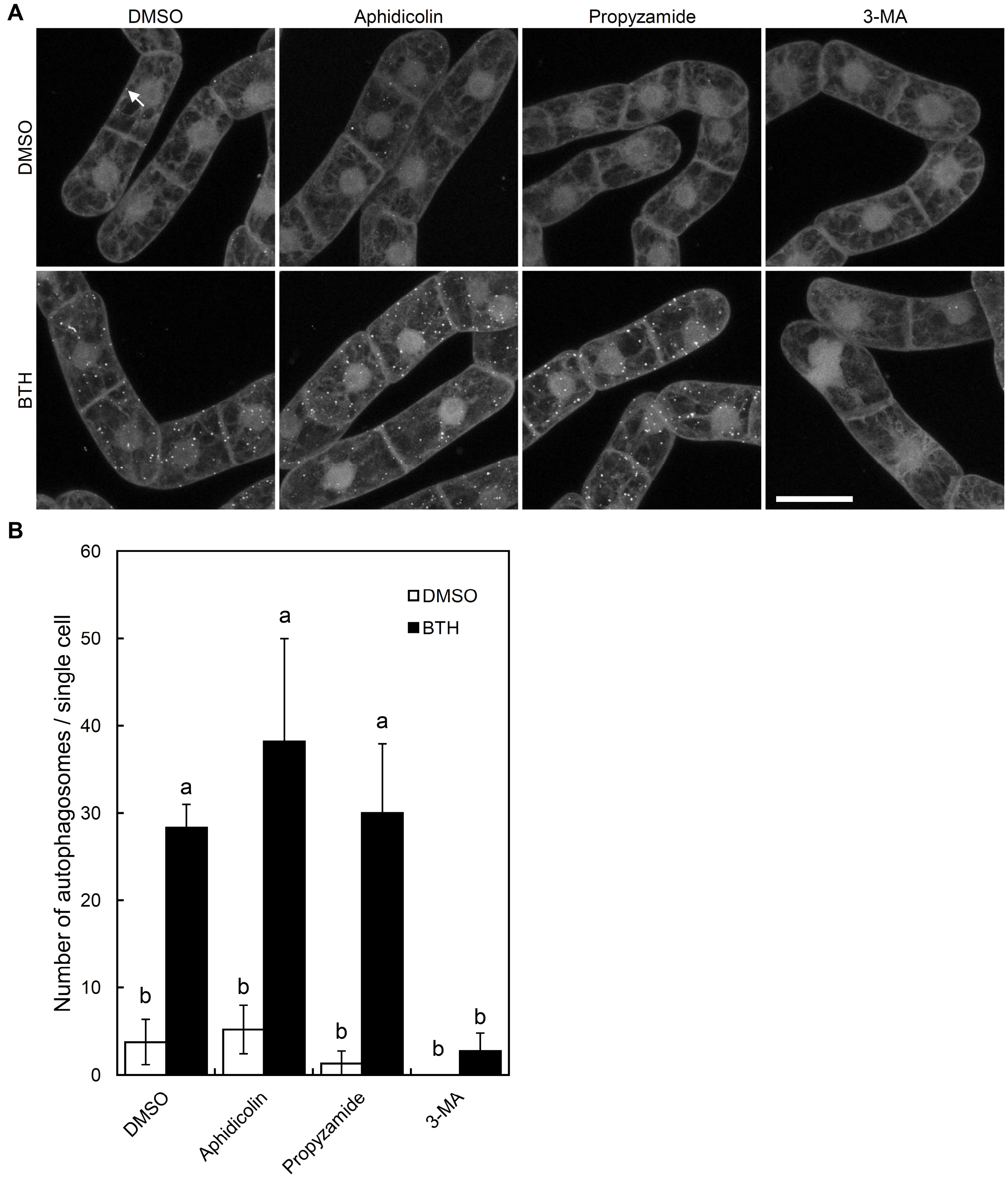

Supplementary Figure S1: The effect of aphidicolin/propyzamide on autophagosome formation in tobacco BY-2 cells. Four-day-old BY-YA8 cells were treated with aphidicolin (5 μg/ml), propyzamide (3 μM), and 3-MA (5 mM) for 3-5 hours each in the presence or absence of BTH (100 μM). The open and closed bars indicate with or without BTH. DMSO was used as control of drugs. 3-MA was used as a well-known inhibitor of autophagosome formation. Cells were observed using CLSM (A), and the number of the punctate signals per single cell was counted (B). Arrows indicate to the punctate signals in YFP-NtATG8a. Scale bars: 50 μm. Data represent a representative experiment from two independent experiments. Data are the means ± SD of 5-7 cells. a, b values with different letters are significantly different ($P < 0.05$).

```
Nt_1 115:LIGGATIHLFNMKKQLKTGKHKLRLWPGKEADGSINTTTPGKVPKEERGELE-RLEKLVN 173
Nt_2 115:LIGGATIHLFNMKKQLKTGKHKLRLWSGKEADGSINTTTPGKVPKEERGELE-RLEKLVN 173
At   114:LIGGATVLLFNSKMQMKSGKQKLRLWQGKEADGSFPTSTPGKVPRHERGELE-RLEKLMN 172
Os   117:IVGGATIFLFNNKKQLKTGRQKLRLWPQKEADGRVPTTTPGKVPKNERGEIE-RLERLVN 175
Hs   121:PVGGTTVSLFGKYGMFRQGMHDLKVWPNVEADGSEPTKTPGRTSSTLSEDQMSRLAKLTK 180
         ..**.*. **. ......*...*..*...****. .*.***.... ...... **..*..
```

Supplementary Figure S2: Amino acid sequence alignment of plant Vps34 proteins with Vps34 of *Homo sapiens*. *Nicotiana tabacum* (Nt) XP_016465630.1 (Nt_1); XP_016473480.1 (Nt_2), *Arabidopsis thaliana* (At) NP_176251.1, and *Oryza sativa* (Os) NP_001054810.1 Vps34 homologs are aligned with *Homo sapiens* (Hs) NP_002638.2, based on their sequence. Residues conserved among Vps34 homologs are colored black. Arrow indicates conserved threonine of Vps34.

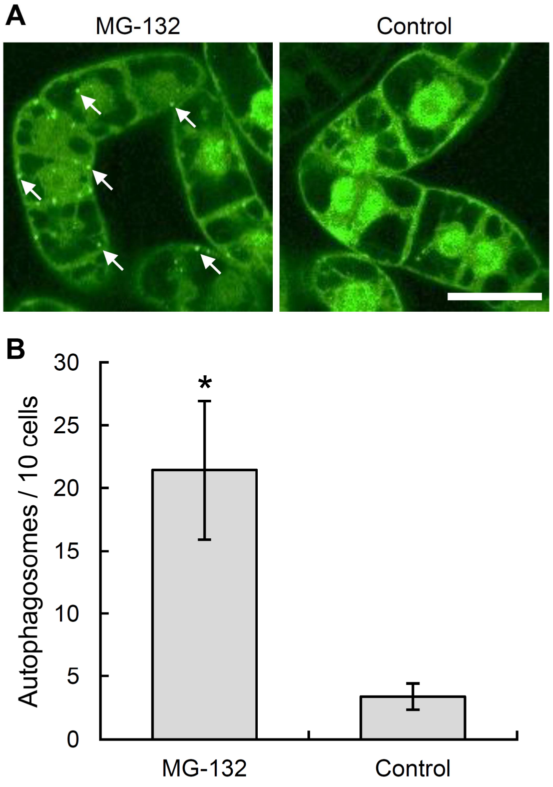

Supplementary Figure S3: The effect of MG-132 on autophagosome formation in tobacco BY-2 cells. Three-day-old BY-YA8 cells were treated with MG-132 (100 μM) for 30 min. DMSO is used as control of MG-132. Cells were observed using CLSM (A), and the number of the punctate signals were counted (B). Arrows indicate to the punctate signals in YFP-NtATG8a. Scale bars: 50 μm. The data are representative of three experiments. Data are the means ± SE of three experiment. * $P < 0.05$, significantly different from the controls.
